# A scATAC-seq atlas of stasis zone in rat skin burn injury wound process

**DOI:** 10.3389/fcell.2024.1519926

**Published:** 2025-01-07

**Authors:** Ruikang Li, Jiashan Li, Shuai Liu, Xinya Guo, Jianyu Lu, Tao Wang, Junjie Chen, Yue Zheng, Yue Yuan, Jiaxin Du, Bolin Zhu, Xiaoyu Wei, Pengcheng Guo, Longqi Liu, Xun Xu, Xi Dai, Runzhi Huang, Xin Liu, Xiaoyan Hu, Shiwei Wang, Shizhao Ji

**Affiliations:** ^1^ Key Laboratory of Resources Biology and Biotechnology in Western China, Ministry of Education, Provincial Key Laboratory of Biotechnology of Shaanxi Province, The College of Life Sciences, Northwest University, Xi’an, China; ^2^ College of Life Sciences, University of Chinese Academy of Sciences, Beijing, China; ^3^ BGI College and Henan Institute of Medical and Pharmaceutical Sciences, Zhengzhou University, Zhengzhou, China; ^4^ Department of Burn Surgery, The First Affiliated Hospital of Naval Medical University, Shanghai, China; ^5^ BGI Research, Hangzhou, China; ^6^ BGI Research, Shenzhen, China

**Keywords:** rat burn injuries, stasis zone, chromatin accessibility, wound healing, transcription factor

## Abstract

Burn injuries often leave behind a “stasis zone”, a region of tissue critically important for determining both the severity of the injury and the potential for recovery. To understand the intricate cellular and epigenetic changes occurring within this critical zone, we utilized single-cell assay for transposase-accessible chromatin sequencing (scATAC-seq) to profile over 31,500 cells from both healthy rat skin and the stasis zone at nine different time points after a burn injury. This comprehensive approach revealed 26 distinct cell types and the dynamic shifts in the proportions of these cell types over time. We observed distinct gene activation patterns in different cell types at various stages post-burn, highlighting key players in immune activation, tissue regeneration, and blood vessel repair. Importantly, our analysis uncovered the regulatory networks governing these genes, offering valuable insights into the intricate mechanisms orchestrating burn wound healing. This comprehensive cellular and molecular atlas of the stasis zone provides a powerful resource for developing targeted therapies aimed at improving burn injury recovery and minimizing long-term consequences.

## 1 Introduction

Burns are a significant global health issue that not only affect patients’ physiological functions but also lead to long-term psychosocial burdens (Jeschke and Herndon, 2014; Radzikowska-Büchner et al., 2023). The repair of skin damage following a burn is a complex process involving interactions among various cell types and molecular signaling pathways (Roshangar et al., 2019). Histologically, the burn area can be divided into three distinct zones: the necrotic zone, which is directly and irreversibly damaged by high temperatures; the stasis zone, which is damaged but has the potential for recovery; and the surrounding normal tissue (Tiwari, 2012; Burgess et al., 2022), which includes the epidermis, dermis, adipose tissue, and muscle layers (Malhotra et al., 2024). The stasis zone is crucial for burn treatment as it represents the transitional area between the wound margin and healthy tissue. With appropriate interventions, this tissue has the potential to be salvaged, thus facilitating the overall healing process (Guo et al., 2021; Lu et al., 2022).

Research indicates that burns heal through epithelial regeneration (Li B. et al., 2021; Boyce and Kagan, 2023), progressing through four stages, hemostasis, inflammation, proliferation, and remodeling (G El Baassiri et al., 2023; Siddique et al., 2023). The management and protection of the stasis zone directly influence inflammatory responses, tissue regeneration, and scar formation during these stages (Guo et al., 2015). First is the early stage after burn, a rapid inflammatory response occurs in the stasis zone, with elevated levels of cytokines such as TNF-α and the IL-1 family in the body. The main cell types involved include monocytes, macrophages, and mast cells, which function in hemostasis, pathogen defense, and necrotic tissue clearance, laying the groundwork for the subsequent proliferation and remodeling phases to promote wound repair (Eming et al., 2007; Burgess et al., 2022). Therefore, the inflammatory response is crucial for balancing apoptosis and proliferation (Ren et al., 2022). As the burn enters the proliferation and remodeling phases, the survival capacity of endothelial cells and fibroblasts significantly increases, primarily to promote angiogenesis and collagen deposition (Huang et al., 2015; Shi et al., 2023). With the formation of granulation tissue at the wound site, epidermal basal cells demonstrate the ability to exceed their self-renewal capacity, gradually differentiating and migrating to the site of injury (Gross-Amat et al., 2020). This process is influenced by growth factors (such as *EGF*, *TGF-β*, *VEGF*, and *FGF*), extracellular matrix components, moisture, and skin appendages (Burgess et al., 2022; Fan et al., 2024). Migrating epithelial cells also facilitate the formation of hair follicles and sweat glands. Ultimately, the proliferative migratory basal cells generate new epithelial cells, restoring the skin’s barrier function after a burn (Sukmawati et al., 2020). Reports indicate that skin injury induces significant changes in the epigenetic mechanisms of the skin, establishing unique epigenetic demands that influence inflammation and tissue repair. Specifically, this involves the production of damage-stress cytokines via epigenetic alterations during inflammation, and the recruitment and differentiation of resident stem cells following skin injury through epigenetic modifications, such as histone methylation and acetylation during re-epithelialization (Plikus et al., 2015; Ran et al., 2023; Rolim et al., 2024). These findings emphasize the complex physiological and biochemical processes involved in burn healing and reveal the importance of chromatin accessibility in regulating the rapid cellular response to injury (Lander et al., 2017; Nascimento-Filho et al., 2020; Cheng et al., 2024). ScATAC-seq data hold promise for providing a more detailed understanding of the role of the stasis zone in burn healing. However, there is still a lack of comprehensive datasets for rat burn.

Our study aims to fill this gap by providing an extensive scATAC-seq atlas of chromatin accessibility in rat skin at various time points following burn injuries. We analyzed 31,503 cells from normal tissue (ctrl) and the burn stasis zone at 0 h (0 h), 12 h (12 h), Day 1 (D1), Day 3 (D3), Day 7 (D7), Day 11 (D11), Day 15 (D15), and Day 19 (D19) post-burn, identifying 26 distinct cell types and their associated regulatory elements. This detailed characterization of chromatin accessibility and cell type-specific gene score will enhance our understanding of the molecular mechanisms underlying burn wound healing and provide valuable resources for future research in mammalian skin regeneration.

## 2 Materials and methods

### 2.1 Tissue collection

**FIGURE 1:**
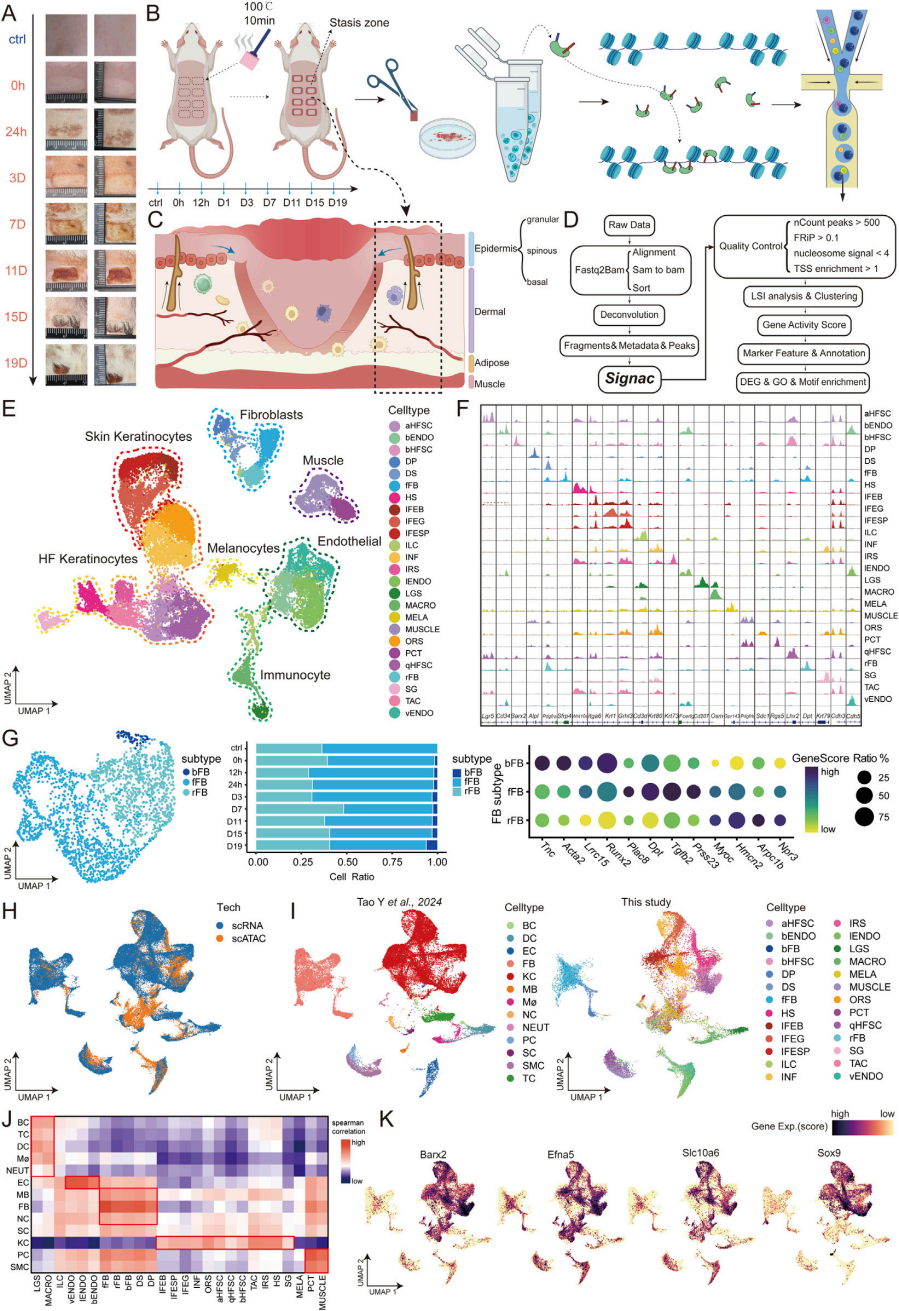
Identification of scATAC data cell types in burn stasis area of rats **(A)** Images of rat burn samples at each time point. **(B)** Experimental workflow: Rat skin samples were collected at nine time points (ctrl, 0h, 12h, D1, D3, D7, D11, D15, D19) for scATAC-seq profiling. **(C)** Diagram of skin burn pattern, including the structure and state of the necrotic area, the stasis area, and the normal skin. **(D)** The analysis workflow for scATAC-seq profiles. **(E)** UMAP visualization of 26 cell types identified by gene score in scATAC-seq data. aHFSC, active hair follicle stem cell; bENDO, burn specific endothelial; bHFSC, burn specific hair follicle stem cells; DP, dermal papilla; DS, dermal sheath; fFB, fascial fibroblast; HS, hair shaft; IFEB, interfollicular epidermis basal; IFEG, interfollicular epidermis granular; IFESP, interfollicular epidermis spinous; ILC, innate lymphoid cell; INF, infundibular; IRS, Inner root sheath; lENDO, lymphatic endothelial; LGS, langerhans; MACRO, macrophage; MELA, melanocyte; MUSCLE, muscle; ORS, outter root sheath; PCT, pericyte; qHFSC, quiescent hair follicle stem cell; rFB, reticular fibroblast; SG, sebaceous gland; TAC, transit amplifying cell; vENDO, vascular endothelium. **(F)** Cell type-specific genome browser views of scATAC-seq signal for the dynamically identified peaks. **(G)** Subpopulations of skin fibroblasts, marker genes, and their proportions over time. **(H)** UMAP shows cell clusters from two datasets. **(I)** Integration of scRNA data from rat skin with radiation injury by Tao Y *et al.* and distribution of cell types in the two datasets. **(J)** Correlation analysis between cell types in this study and public datasets, with red indicating high correlation and blue indicating low correlation. **(K)** FeaturePlot of marker gene expression for bHFSC in the integrated dataset, with darker colors indicating higher expression.

This study was approved by the Affiliated Hospital of the Naval Medical University (license number: SYXK (Hu) 2020–0033) and the BGI Technology Ethics Committee (approval number: BGI-IRB A23034). Experiments were conducted on SPF-grade SD male rats, 8 weeks old. A total of 9 rats were used in the study. In the modeling experiment, a 1 × 2 cm sponge was used to uniformly create 8 burn wounds. At each time point, the stasis zone of two wounds from one rat was collected for scATAC-seq experiments (Figure 1B). The specific modeling steps are as follows: Rats were depilated and prepared the day before the experiment to fully expose the back area. Following anesthesia, the rats were secured on a heated pad. A sponge pad, was heated in boiling water for 10 min, reaching approximately 100°C. The sponge was quickly dried and applied evenly to the rat’s back, ensuring even heating. The burn wound was then covered with gauze and bandaged. Tissue samples from the normal tissue (ctrl) and burn stasis zone were collected at 0h, 12h, D1, D3, D7, D11, D15, and D19 post-burn, preserved in a tissue storage solution, and processed within 48 h (Figures 1A, B).

### 2.2 Dissociation of skin tissue

Skin tissues were placed in PBS (GIBCO), washed, and transferred to a 2 mL tube with 1 mL of enzyme 1 (100 × P/S (XP Biomed), 0.25% trypsin (GIBCO)). The tissue was finely minced with scissors, transferred to a 50 mL tube, and washed with enzyme solution 1. The solution was sealed, placed in a PE glove to prevent leaks, and incubated at 37°C with agitation at 130 rpm for 25 min. The solution was then centrifuged at 500 *g* for 5 min, and the supernatant was carefully removed. Next, 10 mL of enzyme solution 2 (DMEM/F-12 (GIBCO), collagenase IV(BBI), collagenase I (SANGON), dispase II (SIGMA), P/S) was added, mixed, and incubated under the same conditions for 60 min. The cell suspension was filtered through a 70 μm filter, washed with DPBS containing 2% FBS, and filtered again using a 30 μm filter. Cells were centrifuged at 300 *g* for 5 min at 4°C, resuspended in PBS (0.04% BSA (SIGMA)), and assessed for viability using trypan blue staining (INVITROGEN) dye with a Countstar cell analyzer.

### 2.3 scATAC-seq library construction and sequencing

For scATAC-seq library preparation, we used the DNBelab C4 (Yu et al., 2021) Series Single-Cell ATAC Library Prep Set (MGI, #1000012554) to generate 17 libraries across 9 time points, with 2 libraries for each post-burn time point and 1 control. The process involved cell fixation, nuclear extraction, nuclear counting, transposition, droplet generation, emulsion breaking, PCR amplification, purification, fragment selection, circular library construction, and sequencing on the DNBSEQ-T1 platform with read lengths of 115 bp for read 1, 69 bp for read 2, and 10 bp for the sample index sequencing scheme of the China National GeneBank (CNGB) (Huang et al., 2017).

### 2.4 scATAC-seq raw data processing

The scATAC-seq data processing followed these steps: raw reads were demultiplexed into insertions and barcodes, then filtered using PISA (v 1.1) with a minimum sequencing quality of 20. The filtered reads were aligned to the rat genome using BWA (v 0.7.17-r1188) (Li and Durbin, 2009), and BAM files were further processed with bap2 (v 0.6.2) (Lareau et al., 2019) to assign barcodes to individual cells.

### 2.5 Quality control (QC) of the scATAC-seq downstream analysis

Low-quality cells were filtered using Signac (v1.6.0) (Stuart et al., 2021) with the following criteria: nCount peaks >500, fraction of reads in peaks (FRiP) > 0.1, nucleosome signal <4, and transcription start site (TSS) enrichment >1 (Figure 1D). Doublets were identified and removed using the scDblFinder (v1.12.0) package.

### 2.6 Integration and clustering of scATAC-seq data

In Signac (Stuart et al., 2021), use the *reduce* function to merge multiome peaks sets from scATAC-seq libraries. LSI dimensionality reduction was performed with the *RunSVD* function, and sample integration was done using the *IntegrateEmbeddings* with Canonical Correlation Analysis (CCA). Clustering was carried out with *FindClusters* function at a resolution of 0.7, and cluster-specific peaks were identified using the *FindAllMarkers* function.

### 2.7 scATAC-seq gene activity scores

Gene activity scores were computed using Signac’s *GeneActivity* function, with the RNA matrix created using the *CreateAssayObject* function. Due to the sparsity of scATAC-seq data, we employed MAGIC (Markov Affinity-based Graph Imputation of Cells) (van Dijk et al., 2018) for data smoothing and imputation by constructing an affinity graph and applying Markov diffusion.

### 2.8 Gene ontology (GO) enrichment analysis

GO enrichment analysis was conducted on gene sets for each cell type using the *enrichGO* function in the clusterProfiler (v 4.6.0) (parameters: OrgDb = org. Rn.e.g.,.db, ont = ‘ALL’) (Wu et al., 2021). Input genes (p < 0.01, log2FC > 0.5) were obtained from the gene activity matrix using *FindAllMarkers*.

### 2.9 Motif enrichment analysis

Motif enrichment was performed with Signac’s *getMatrixSet* function (tax_group = “vertebrates”) to retrieve motif position frequency matrices. The *AddMotifs* function (genome = “BSgenome.Rnorvegicus.UCSC.rn7”) was used to add motif information, and a heatmap was generated to display the top 4 of cell type-specific motifs.

### 2.10 Soft clustering of peaks varying with burn time points

To perform clustering analysis using Mfuzz (v 2.58.0) (Kumar and E Futschik, 2007), first input the peak matrix, where rows represent different peaks and columns represent different time points or cells. Use the *standardise* function to standardize the data, then estimate the fuzzification parameter (m) with the *mestimate* function. Next, execute the fuzzy C-means clustering analysis with the *mfuzz* function. The clustering results indicate the temporal trends of each peak.

### 2.11 Monocle 2 pseudotime analysis

Analyzing the differentiation trajectory of bHFSC and the genes influencing the trajectory using monocle2 (v2.22.0) (Qiu et al., 2017). The top specific peaks calculated by the Seurat *FindAllMarkers* function will be input into monocle2 to identify cell differentiation trajectories. After selecting the keratinocyte lineage, dimensionality reduction will be performed using the DDRTree method, and visualized with the *plot_cell_trajectory* function, followed by monocle2 pseudotime analysis. To identify peaks influencing the generation of the IFE lineage from bHFSC, the *BEAM* function will be used to identify lineage branch-enriched peaks (*P*-value < 0.01). Peaks will be annotated to genes using the *annotatePeak* function within regions 2 kb upstream and downstream of the transcription start site (TSS)., the chromatin accessibility of relevant genes along the lineage will be visualized using ComplexHeatmap (v2.14.0) (Gu et al., 2016).

## 3 Results

### 3.1 scATAC-seq quality control across burn time points

In this study, we collected samples of normal skin and stasis zone tissues from rats at nine time points, spanning from 0 h to 19 days post-burn. Nuclei were extracted from these tissues for scATAC-seq experiment. The raw sequencing data were processed through alignment and deconvolution to generate fragments, metadata, and peak files, yielding an average of over 23 million scATAC-seq reads per sample. Quality control was performed using the Signac workflow, with the following thresholds: nCount peaks >500, FRiP >0.1, nucleosome signal <4, and TSS enrichment >1 (see Methods). High-quality data were obtained, with each sample containing an average of 3,500 cells. Additionally, the mean unique cell fragments per sample were 11,623, and the average TSS enrichment was 3.78 (Supplementary Table S1), confirming a significant concentration of reads around TSS regions (Buenrostro et al., 2013) (Supplementary Figures S1A–C). To ensure purity, doublets were removed from each library using *scDblFinder* (Supplementary Figure S1D). The final dataset comprised 31,503 single cells, offering a comprehensive chromatin landscape of stasis-zone tissues after burn injury.

### 3.2 Comprehensive chromatin accessibility map revealing multiple cell types in burned skin and its appendages

Data from different time points were effectively integrated, followed by visualization with Uniform Manifold Approximation and Projection (UMAP), revealing the heterogeneity and unity of cell types across various burn injury time points (Supplementary Figures S1E, F). Our chromatin accessibility map of burned skin identified 26 distinct cell types and their subtypes within the epidermis, dermis, and skin appendages (Figures 1E–G; Supplementary Table S2). Starting with the epidermis, a critical layer of the skin, we identified various cell types, including basal cells of the interfollicular epidermis (IFEB) (*Itga6, Tp63, Wn3*), granular cells (IFEG) (*Krt1, Spink5, Krt10*), and spinous cells (IFESP) (*Grhl3, Pkp1, Dsc3*) (Mazzone et al., 2016; Ågren et al., 2022). Specialized cells within the epidermis include melanocytes (MELA) (*Gpr143, Tyr, Mlana*) (Carney et al., 2024), which are essential for pigmentation, and sebaceous gland cells (SG) (*Krt79, Acsbg1*) (Lanz et al., 2011), which contribute to lipid production. Epidermal lineage-related hair follicle stem cells, including quiescent (qHFSC) (*Lhx2, Krt15, Itgb8*), activated (aHFSC) (*Lgr5, Itgb8*), and burn-specific states (bHFSC) (*Barx2, Itgb8*) (da Silva-Diz et al., 2013), were also identified. In the dermal compartment (Figures 1E, F), we identified multiple fibroblast populations, fascial fibroblasts (fFB) (*Sfrp4, Prss23, Tgfb2*), reticular fibroblasts (rFB) (*Dpt, Npr3, Arpc1b*) and burn-specific fibroblasts (bFB) (*Tnc, Acta2, Lrrc15*) (Figure 1G) (Gay et al., 2020; Correa-Gallegos et al., 2023). The hair follicle structures within the dermis include dermal papilla (DP) (*Alpl, Col23a1, Runx3*), dermal sheath (DS) (*Pdgfra, Entpd1, Fbn1*) (Kim et al., 2024). The hair shaft (HS) (*Wnt10a, Wnt3, Krt27*) and surrounding layers, such as the inner root sheath (IRS) (*Krt73, Krt25, Krt27*) and outer root sheath (ORS) (*Sdc1, Krt17*), were also detected. Additionally, infundibular cells (INF) (*Krt80, Krt17, Itgb8*)and transit amplifying cells (TAC) (*Cdh3, Lef1, Bmp7*) play key roles in hair follicle biology, while muscle cells (MUSCLE) (*Pdgfrb, Mylk, Rgs5*) and pericyte (PCT) (*Rgs5,Tagln*) reflect the multifunctionality of the skin (Takahashi et al., 2020). The immune system within the skin includes innate lymphoid cells (ILC) (*Cd3d, Ptprc, Runx3*), macrophages (MACRO) (*Osm, Itgax, Cd86*) (Wang et al., 2019), and Langerhans cells (LGS) (*Cd207, Itgax*) (Hochgerner et al., 2022). Finally, the vascular and lymphatic systems were represented by burn-specific endothelial cells (bENDO) (*Pecam1,Cd34,Aqp1*), lymphatic endothelial cells (lENDO) (*Fcerlg, Il1r1, Flt4*), and vascular endothelium (vENDO) (*Cdh5, Cd93, Pecaml*) (Figure 1F; Supplementary Figure S2A; Supplementary Table S2) (Hou et al., 2020; Li et al., 2020). As burn injury progresses over time, there is a significant shift in the proportion of various cell types. For instance, MACRO and IENDO cells dominate the early stages at 0h and 12 h post-burn, which is likely associated with the inflammatory response in the stasis zone. Conversely, the proportions of other cell types such as vENDO and the hair follicle components HS, IRS, and ORS decrease at 0h and 12 h post-burn, potentially due to cell death caused by the burn injury. Subsequently, the ratios of vENDO, qHFSC, and the stratified cells of IFE gradually increase, which may indicate the process of tissue regeneration and remodeling (Supplementary Figure S1G). This diverse cellular composition reflects the complex structural and function of the skin and its response to burn injury.

### 3.3 Integration and comparative analysis of cell types in skin burn and radiation injury healing processes

Our dataset was integrated with the public scRNA-seq dataset of radiation-induced skin injury (GSE193564). Single-cell RNA-Seq analysis of molecular changes during radiation-induced skin injury: the involvement of Nur77 and various cell types were well integrated (Figures 1H, I). We reproduced the cell type-specific marker genes provided in the public article for annotation (Supplementary Figure S1F, I), and the results showed good consistency between our cell type annotations and the public data. Next, we calculated the Spearman correlation between the two datasets and compared the RNA matrices of cell types from both datasets. The results showed that the cell types mainly fell into five major categories: immune cells, endothelial cells, fibroblasts, keratinocytes, and muscle cells, all of which exhibited high correlation (Figure 1J). Additionally, the openness of marker genes *Barx2, Efna5, Slc10a6*, and *Sox9* for bHFSC in our dataset showed consistent trends with their expression in the scRNA data (Figure 1K) (Tierney et al., 2024). In summary, the chromatin accessibility revealed by our scATAC-seq data showed a high correlation with the mRNA expression of the same cell types in the public scRNA-seq data, demonstrating the completeness of our burn dataset time points and the high reliability of our data.

However, some differences were also observed. For example, the bENDO and MELA cell types we identified were rarely found in the radiation injury dataset (Figure 1I). By comparing sampling photos of the two injury types, we found that the radiation injury site showed signs of healing after 19 days, while the burn stasis zone had fully recovered, but the necrotic area was still scabbing (Figure 1A) (Yan et al., 2024). This suggests that burns may cause a more intense inflammatory response and prolong the inflammation phase, leading to an increase in bENDO cells, which delays the proliferation and remodeling phase of the skin and may result in more pigment deposition during burn healing (Burgess et al., 2022).

### 3.4 Gene functions and motif enrichment analysis reveals key transcription factors in burn skin cell types

**FIGURE 2:**
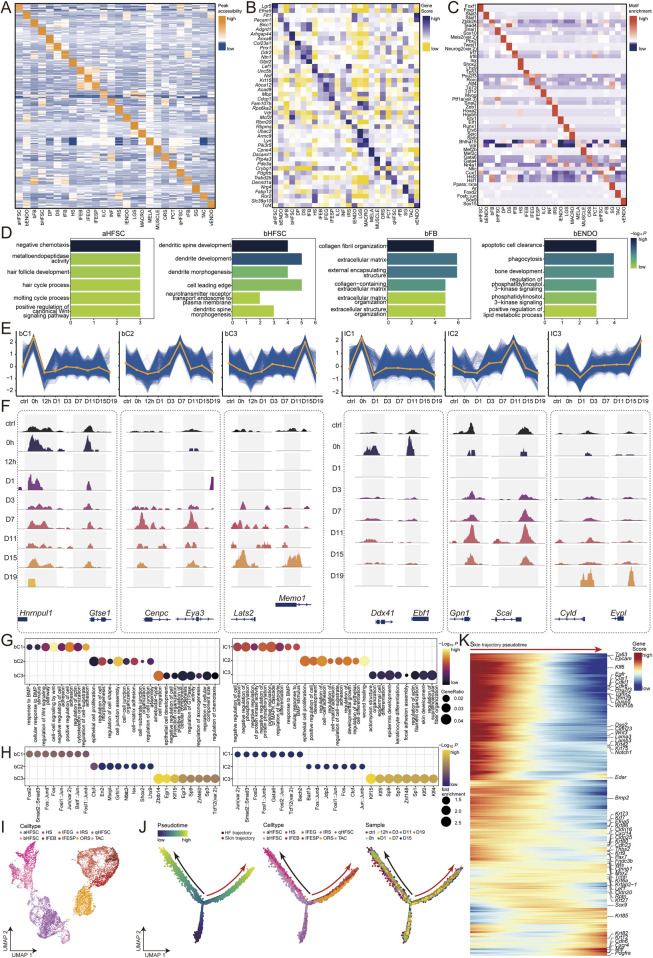
Temporal and pseudotime analyses of gene regulation, cell type dynamics, and the integration of single-cell RNA data. **(A)** Cell type-specific peaks. **(B)** Cell type-specific genes. **(C)** Cell type-specific motifs. **(D)** Enriched Gene Ontology (GO) Terms for aHFSC, bHFSC, bFB and bENDO. **(E)** Standardized fuzzy clustering of scATAC signals for bHFSC and IFEB, with blue lines representing individual loci and orange lines representing cluster center values. **(F)** Genomic browser view of dynamic peaks of bHFSC and IFEB signals. **(G)** GO enrichment of clusters for bHFSC and IFEB, with dot size representing the proportion of genes in the GO term and color representing the -Log10 (*P*-value). **(H)** Motif enrichment of clusters for bHFSC and IFEB, with dot size representing the fold. enrichment and color representing the -Log10 (*P*-value). **(I)** UMAP of cell types constructed in pseudotime. **(J)** Pseudotime analysis of post-injury epithelial lineage of HFSC and normal hair follicle differentiation using Monocle2. **(K)** Peaks associated with burn-specific epithelial temporal changes will be annotated to genes, with red coloration indicating high scores and blue coloration indicating low scores.

To explore the underlying mechanisms of chromatin accessibility profiles, we performed peak differential analysis, gene scoring, and motif enrichment analysis on 163,441 peaks. We identified potential cis-regulatory elements (Figure 2A), transcription factors (TFs), genes, and their functions closely associated with the 26 cell types. For example, qHFSC are enriched for cell proliferation and adhesion-related functions (Supplementary Figure S2B). The gene Trabd2b is involved in regulating the Wnt signaling pathway, which controls stem cell self-renewal (Figure 2B). Additionally, Cux1 motifs are highly enriched, maintaining the balance of cell proliferation and differentiation in epithelial tissues (Figure 2C) (Ren et al., 2023). aHFSC are enriched for functions involved in hair follicle development and hair growth cycle (Figure 2D). The gene *Lgr5* is involved in maintaining hair follicle stem cell self-renewal (Figure 2B) (da Silva-Diz et al., 2013). Furthermore, Fox family motifs (Foxf1, Foxp1) are enriched, indicating their active proliferation to support follicle growth. (Yang et al., 2022). The Fox family genes are known to play crucial roles in stem cell maintenance, differentiation, and follicle cycle regulation (Figure 2C) (Wang et al., 2016). bHFSC are associated with cell movement and neurotransmitter transmission (Figure 2D). The gene *Anxa8* related to dynamic changes in the cell membrane and cell migration (Figure 2B) (Yuan et al., 2021), with the Snai1 motif regulating cell migration and survival (Figure 2C), bENDO cells are enriched for functions related to the clearance and phagocytosis of apoptotic cells (Figure 2D). The gene *Snai1* enhances cell anti-apoptotic ability and promotes cell survival (Figure 2B) (Freihen et al., 2020). With enrichment for STAT family motifs (Stat3, Stat1). Stat3 promotes cell survival, proliferation, and differentiation by activating growth-promoting and apoptosis-inhibiting genes (Chou et al., 2021), while Stat1 is involved in regulating immune responses (Figure 2C) (Xu et al., 2019). bFB cells are enriched for functions related to collagen fiber tissue remodeling (Figure 2D). The gene *Bicc1* plays an important role in fibrosis in the kidney and other tissues (Figure 2B) (Leal-Esteban et al., 2018), promoting the generation of myofibroblasts, with enrichment for Tead4 motifs, which bind to TEA/ATTS elements on DNA and activate genes such as α-smooth muscle actin (α-SMA) (Figure 2C) (Mesrouze et al., 2017). These results list the key motifs associated with burn skin remodeling, including both previously reported and newly discovered burn-specific TFs. This demonstrates the comprehensiveness and accuracy of our cell type data, strongly suggesting that the scATAC-seq profiles of burn rats precisely identify accessible chromatin regions within the rat genome, providing valuable data resources and theoretical foundations for studying mammalian burn healing.

### 3.5 Temporal dynamics of chromatin accessibility during burn wound healing

The skin samples collected from the burn stasis zone of rats from 0 to 19 days post-burn constitute a comprehensive scATAC-seq dataset (Figure 1A), which can elucidate which cell types are most sensitive to burns and the molecular mechanisms behind healing. We found that the cell types in the skin niche mainly exhibit two trends with the change in burn time points: one increases in the early stages of the burn, producing burn stress responses and immune responses, including bHFSC, MACRO, MELA, fFB, and PCT. The other increases in the later stages of the burn, participating in skin remodeling, including IFEB, IFESP, IFEG, and ORS (Supplementary Figure S3A). To explore the molecular mechanisms of epithelialization after burns, we focused on bHFSC, which increases in the early stages, and IFEB, which increases in the later stages. We performed normalized time point fuzzy clustering on peaks and selected three significant trends for each cell type, showing the categories at different stages of burn healing (Supplementary Tables S4, S5): (1) bHFSC cluster 1 (bC1, n = 11773) accessible immediately after injury at 0h, indicating stress response; (2) bHFSC cluster 2 (bC2, n = 8,844) accessible during stem cell differentiation and proliferation from D3-D11; (3) bHFSC cluster 3 (bC3, n = 8,568) accessible during skin remodeling from D11-D19; (4) IFEB cluster 1 (IC1, n = 11399) accessible immediately after injury at 0h, indicating cell damage response; (5) IFEB cluster 2 (IC2, n = 10574) accessible during epithelial cell proliferation from D7-D15; (6) IFEB cluster 3 (IC3, n = 3,516) accessible during skin remodeling from D15-D19 (Figure 2C). These clusters highlight the chromatin changes during wound healing, with burns causing rapid cell damage and immune responses, clearing necrotic cells to prepare for later skin healing. The dynamics of chromatin accessibility provide new insights into cell fate determination during burn healing.

We identified significantly changed chromatin accessibility peaks in response to injury stress from bC1. By associating them with genes, we found that they mainly play roles in functions related to BMP and Wnt signaling pathways (Figure 1G). For example, *Gtse1* is involved in cell cycle regulation and DNA damage response, affecting the cells response to signals (Lei et al., 2020). *Hnrnpul1* can influence the cells response to DNA damage by regulating the function of the p53 protein (Figure 1F) (Sharma et al., 2015). The enriched motif is a heterodimer composed of Fos and Jun family proteins, which can respond to cellular stress and regulate gene expression (Figure 1H) (Papachristou et al., 2006). bC2 describes the roles of cells in epithelial cell proliferation, morphogenesis, cell junction, and adhesion regulation. For example, the *Cenpc* gene encodes centromere protein C, which is involved in chromosome segregation and cell division (Figure 1G) (Fellmeth et al., 2023); the *Eya3* gene encodes a transcription factor that plays an important role in cell development and differentiation (Figure 1F) (Wiedner et al., 2023). Grhl1 and Nfatc3 motifs are involved in these processes (Figure 1H) (de la Garza et al., 2013). bC3 functions in migration, epithelial cell development, GTPase activity regulation, and cell component size regulation (Figure 1G). For example, the *Lats2* gene encodes a protein that is a key regulator of the Hippo signaling pathway, involved in the regulation of cell proliferation, apoptosis, and morphogenesis (Guo et al., 2019); the *Memo1* gene encodes a protein that plays an important role in cell migration and signal transduction (Figure 1F) (Labrecque et al., 2021). Gr1 and Egr3 motifs are involved in the regulation of cell migration and epithelial cell development (Figure 1H) (Shaw et al., 2015; Nishimura et al., 2024). IC1 functions in the regulation and response to cell death and immune response (Figure 1G). For example, the *Ddx41* gene encodes a protein involved in RNA metabolism and innate immune response (Kim K. et al., 2023); the *Ebf1* gene encodes a transcription factor that plays an important role in B cell development and differentiation (Figure 1F) (Kim E. E. et al., 2023). Bach2 and Gata6 motifs are involved in the regulation of cell death and B cell development and immune response (Figure 1H) (Jayakumar et al., 2022; Weng et al., 2024). IC2 describes the regulatory functions of cells in epithelial cell proliferation, development, adhesion, differentiation, and wound healing (Figure 1G). For example, the *Gpn1* gene encodes a protein involved in the nuclear localization and stability of RNA polymerase, with its main function related to transcription regulation (Huang et al., 2021); the *Scai* gene encodes a protein that plays an important role in cell migration and invasion, particularly by regulating the cytoskeleton and adhesion molecules (Figure 1F) (Yi et al., 2019). Fos::Jund, Fos, Fosl1::Jun, and Jun::Junb motifs are involved in the regulation of epithelial cell proliferation, development, adhesion, differentiation, and wound healing (Figure 1H) (Sarate et al., 2024). IC3 describes the remodeling functions of cells in actin-myosin structure, cell junction, epithelial cell differentiation, and skin development (Figure 1G). For example, the *Cyld* gene encodes a deubiquitinating enzyme involved in regulating cell signaling, cell cycle, and apoptosis (Li Y. et al., 2021); the *Evpl* gene encodes a structural protein in the epidermis and other epithelial tissues, involved in the organization of the cytoskeleton and stabilization of cell junctions (Figure 1F) (Laky et al., 2023). Klf5 and Klf4 motifs are involved in the regulation of cell junction stabilization and epithelial cell differentiation (Figure 1H) (Laky et al., 2023). In summary, our comprehensive analysis of chromatin accessibility dynamics in response to burn injury provides valuable insights into the molecular mechanisms and cellular processes involved in wound healing, highlighting the distinct roles of various cell types and signaling pathways at different stages of the healing process.

### 3.6 Putative cell lineage transition in stasis zone of burn healing skin

The skin contains various epidermal stem cell populations, among which HFSC are crucial for the cyclical growth of hair (Kloepper et al., 2015). Previous studies have reported that HFSC undergo a change in stem cell identity and mobilize to participate in epidermal repair when the skin is scratched or abraded (Tierney et al., 2024). However, little is known about the lineage changes of HFSC during the burn process (Babakhani et al., 2020). To investigate the relationship between HFSC and burn skin healing, we applied the Monocle2 algorithm to scATAC-seq data to predict the fate transitions of HFSC after burns (Qiu et al., 2017). Our analysis clearly revealed the burn-specific epithelialization lineage and the lineage of HFSC in normal hair follicle growth. Specifically, we found that qHFSC transition into bHFSC after burns, subsequently producing IFESP and IFEG through IFEB. Additionally, we predicted that qHFSC activate into aHFSC in normal conditions, which then differentiate into ORS and TAC, with TAC further generating IRS and HS. (Figures 2I, J).

In the proliferation, differentiation, and formation of basal cells of the skin after burns, which subsequently generate the spinous and granular layers, the following genes play key roles: First, during the proliferation of qHFSC, stem cell genes with high gene scores, such as *Tp63, Klf6, Pou2f3, and Tgfbr2* are crucial in maintaining stem cell characteristics (Paris et al., 2012; Tomela et al., 2021; Li et al., 2022). Subsequently, during the transition to bHFSC, stem cell and cell migration genes with high gene scores, such as *Dsg2, Cldn23, Wnt3*, and *Notch1* promote cell proliferation and migration (Kishimoto et al., 2000; Brennan et al., 2007). Next, bHFSC differentiate into IFEB, basal layer genes with highly gene scores, such as *Bmp2, Krt7, Krt73, Krt80, Krt9, and Pax7* playing important roles in basal cell differentiation (Gosselet et al., 2007; Kim et al., 2016). Following this, basal cells continue to proliferate and begin to differentiate into the spinous and granular layers, keratinization genes with high gene scores, such as *Krt6a, Krtap2-1, Krt27, Krt85, Krt82*, and *Krt12* being crucial for the formation and maturation of the keratin layer (Suárez-Fariñas et al., 2015; Morgan et al., 2020; Erjavec et al., 2022). Additionally, transcription factors such as Lef1, Msx2, Sox9, and Mitf also play regulatory roles in this process, coordinating cell differentiation and keratin layer formation (Figure 2K) (Yeh et al., 2009; Phan et al., 2020; Wang et al., 2022). Our lineage analysis uncovered the time point-specific transitions and changes in chromatin accessibility during the burn process.

## 4 Conclusion

This study identified 26 different cell types, including various cell types in the epidermis, dermis, and skin appendages, and analyzed their dynamic changes during the burn process. By integrating and comparing burn data with scRNA-seq data of radiation injury, it was found that burns may cause a more intense inflammatory response and prolong the inflammation phase, leading to an increase in bENDO cells, delaying the skin proliferation and remodeling phases, and potentially resulting in more pigment deposition during burn healing. Through motif enrichment analysis, key transcription factors and gene functions related to burn skin cell types were identified, such as cell adhesion, Epidermal development, cell movement, phagocytosis, and collagen fiber tissue remodeling. The study revealed dynamic changes in chromatin accessibility after burns, indicating that burns cause rapid cell damage and immune responses, clearing necrotic cells to prepare for subsequent skin healing. The study predicted the fate transitions of HFSCs in normal hair follicle growth and burn-specific epithelialization lineages, revealing time point-specific transitions and changes in chromatin accessibility during the burn process. These findings provide important theoretical foundations and data resources for understanding the molecular mechanisms of burn skin repair and offer new insights for developing targeted therapies for burn treatment.

## Data Availability

The datasets presented in this study can be found in online repositories. All raw data have been submitted to the CNGB (Accession ID, CNP0006143).
