# Supplementary material for: A scATAC-seq atlas of stasis zone in rat skin burn injury wound process
Source: Front Cell Dev Biol. 2025 Jan 7;12:1519926. doi: 10.3389/fcell.2024.1519926 (PMC11752905; doi:10.3389/fcell.2024.1519926)
Supplement: Supplementary file 1 [file DataSheet3.docx]

Supplementary Material

# Supplementary Data

Supplementary File 1: Creating seurat objects, quality control and integration

Supplementary File 2: Annotation, differential analysis, GO and motif analysis

Supplementary File 3: Mfuzz and pseudotime analysis

Supplementary File 4: Integration with public data

Supplementary File 5: Helper functions

# Supplementary Figures and Tables

## Supplementary Tables

Supplementary Table 1. scATAC-seq metadata and mapping statistics.

Supplementary Table 2. Cell type-specific marker genes list.

Supplementary Table 3. Cell type DEG GO

Supplementary Table 4. bHFSC Mfuzz cluster

Supplementary Table 5. IFEB Mfuzz cluster

## Supplementary Figures

**Supplementary Figure 1. Quality control of scATAC-seq data**

**(A)** Violin plot showing log_10_(uniqueFrags) distribution for each sample. **(B)** Violin plot illustrating TSS.enrichment scores across samples. **(C)** Density plot of TSS.enrichment cutoff values used for QC at nine time points (ctrl, 0h, 12h, D1, D3, D7, D11, D15, D19). **(D)** Scatter plot displaying doublet scores across nine sampling time points, highlighting cells with higher doublet scores in yellow. **(E)** UMAP visualization of data from nine time points, color-coded to distinguish each sampling period. **(F)** UMAP plots depicting separation of data from nine sampling time points. **(G)** Histogram of cell proportion stacking at different burn time points. **(H)** The t-SNE plot displays public scRNA-seq data cell clusters with combined groupings. Each dot represents a single cell. B cells, BC; Dendritic cells, DC; Endothelial cells, EC; Fibroblasts, FB; Keratinocytes, KC; Macrophage, Mφ; Myoblasts, MB; Neural cells, NC; Neutrophil, NEUT; Pericytes, PC; Schwann cells, SC; Smooth muscle cells, SMC; T cells, TC. **(I)** Dot plot illustrating the expression of representative genes for each cell type in Supplementary Figure 1H.

**Supplementary Figure 2. UMAP of cell marker scores and GO cell types**

**(A)** UMAP visualization of cell type marker gene showed that yellow expression was high. **(B)** Enriched Gene Ontology (GO) Terms for cells other than those in Figure 1G.

**Supplementary Figure 3. Supplementary visualization of pseudotime and public data**

**(A)** In the stasis zone of burn injury, a line chart depicting the changes in cell proportion over time points, the early increased cell types are bHFSC, fFB, MACRO, MELA, and PCT, and the late increased cell types are IFEB, IFESG, IFESP, and ORS. **(B)** The pseudotime trajectory in Figure 2G, with the middle panel split to display each color representing a distinct cell type.
